# Screening for anti-adipogenic, pro-lipolytic and thermogenic plant extracts by models associating intestinal epithelial cells with human adipose cells

**DOI:** 10.1007/s00394-021-02794-8

**Published:** 2022-01-29

**Authors:** Damien Guillemet, Chloé Belles, Aurélie Gomes, Vincent Azalbert, Mathilde André, Nourdine Faresse, Rémy Burcelin, Jean-Michel Lagarde, Danièle Lacasa, Mayoura Kéophiphath

**Affiliations:** 1 Nexira, 129 chemin de Croisset, 76000 Rouen, France; 2 Centre Pierre Potier, D.I.V.A. Expertise, 1 place Pierre Potier, 31100 Toulouse, France; 3 Centre Pierre Potier, Imactiv 3D, 1 place Pierre Potier, 31100 Toulouse, France; 4 Institute of Cardiovascular and Metabolic Diseases, 1 avenue du Professeur Jean Poulhès, 31432 Toulouse, France

**Keywords:** Plant extracts, Adipose cells, Caco-2, Lipid accumulation, UCP1, Energy metabolism, Lipolysis

## Abstract

**Purpose:**

Excessive fat mass accumulation in obesity leads to diverse metabolic disorders, increased risks of cardiovascular diseases and in some cases, mortality. The aim of this study was to screen the actions of botanical extracts intended for oral use on human adipose tissue, using an in vitro screening model combining human intestinal cells with human adipose cells. This was to find the most effective extracts on lipid accumulation, UCP1 expression and ATP production in pre-adipocytes and on adipocyte lipolysis.

**Methods:**

In this study, 25 individual plant extracts were screened for their effects on human adipose cells. Consequently, an original in vitro model was set up using the Caco-2 cell line, to mimic the intestinal passage of the extracts and then exposing human adipose cells to them. The biological actions of extracts were thus characterized, and compared with a coffee extract standard. The most effective extracts, and their combinations, were retained for their actions on lipid accumulation, the expression of the thermogenic effector UCP1 and ATP production in pre-adipocytes as well as on lipolysis activity of mature adipocytes.

**Results:**

The biphasic culture system combining human Caco-2 cells with human adipose cells was verified as functional using the green coffee extract standard. Out of the 25 plant extracts studied, only 7 and their combinations were retained due to their potent effects on adipose cells biology. The data showed that compared to the coffee extract standard, Immortelle, Catechu, Carrot and Rose hip extracts were the most effective in reducing lipid accumulation and increased UCP1 expression in human pre-adipocytes.

**Conclusion:**

This study reveals the potential inhibitory effects on lipid accumulation and thermogenic activity of Immortelle, Catechu, Carrot and Rose hip extracts, and for the first time synergies in their combinations, using an in vitro model mimicking as closely as possible, human intestinal passage linked to adipose cells. These findings need to be confirmed by in vivo trials.

**Supplementary Information:**

The online version contains supplementary material available at 10.1007/s00394-021-02794-8.

## Introduction

According to the World Health Organization, the prevalence of overweight and obesity in 2016, was 39% of adults [1]. The burden of morbidity from obesity is strongly linked to cardiovascular and chronic inflammatory diseases [2]. Excessive accumulation of adipose tissue (AT) triggers systemic low-grade inflammatory pathways through local immune cells [2–5], hence, representing a major target for research in obesity and associated metabolic diseases [6]. The increase of AT mass is due to hypertrophy (increase in adipocyte size, i.e., lipogenesis) and hyperplasia [7, 8]. Hypertrophic expansion of mature adipose cells has been shown to promote insulin resistance and other obesity-associated metabolic complications and to be a consequence of inability to recruit new adipose progenitors. Hyperplasia (or adipogenesis) is a fundamental process of increased adipocyte number, and thus of tissue growth, by maturation of pre-adipocytes from an established pool during childhood and would be more protective than deleterious regarding obesity-associated metabolic disorders [9].

AT constitutes different adipocyte types, each with specific functions. While white adipocytes are specifically dedicated to the control of triglycerides and fatty acid storage and fluxes, brown adipocytes have a thermogenic activity which consists of the production of body heat through increased adipocyte metabolism [10]. Interestingly, beige adipocytes share common activities with both white and brown adipocytes. Hence, in the context of early-stage comorbid obesity, AT modulation and specifically, the stimulation of beiging and thermogenesis through UCP1 expression, has now become a target of choice [11–13] and needs to be studied to elucidate preventive and therapeutic strategies, with the additional advantage of not having lipids released into the blood stream.

Accessibility of any targeted tissue to orally administered products, requires the consideration of the different steps involved. Pharmacokinetic evaluation is defined by the combination of the processes of liberation (compounds released in the gut lumen), absorption (plasmatic or blood uptake kinetics), metabolization (chemical conversion by host cells) and excretion. In addition, other processes are also implicated in the bioavailability of products taken orally, such as their distribution to specific tissues, pharmacodynamics (drug and other compound interactions) as well as the more recently studied, metabolization by microbiota.

However, to be able to investigate in detail these processes in vitro, models which mimic as closely as possible these steps, have been or need to be set up. Regarding the simulation of the gastrointestinal tract, models dedicated to the investigation of colic metabolism, models such as Shime^®^ [14] and that for studying digestion in the upper intestinal tract (gastric and the small intestine), Tim^®^ [15], have already been set up and used. Though, these models have the limitation of compound absorption and metabolization by the intestinal cells and are moreover, not really adapted to high-throughput screening.

Then again, other in vitro models focus mainly on epithelial cell-like cultures, especially in contiguous monolayer systems to simulate the intestinal mucosal barrier. In addition, Caco-2 cells differentiated into a monolayer have an enterocyte-like phenotype and are the primary cellular model used to mimic the human intestinal epithelium, with the apical side facing the intestinal lumen and the basolateral side facing the connective tissue [14]. Caco-2 cells also express, efflux proteins, and enzymes capable of metabolizing molecules to ensure the chemical conversion of test substances. That is why Caco-2 cells have widely been used to quantify trans-epithelial transport, using radio-tracers or tracers labeled with stable isotopes, which can thereby be detected using LC or GC-MS [15].

In previous studies, in vitro models were developed to determine the relationship between Caco-2 cells and adipose biology in co-culture systems. Ishihara R. et al. showed that Caco-2-derived secretions modulate the secretion of the two major adipokines, namely Leptin and Adiponectin, by immortalized rodent-derived adipocyte cells [16]. The bi-directional cellular relationship has also been demonstrated, by Wu and colleagues, showing that the inflammatory state of cultured adipocytes influenced the tightness of Caco-2 cell monolayers [17]. More recently, an in vitro multi-culture model, allowing the measurement of intestinal absorption using adipocytes has been described. In this model, immortalized murine pre-adipocytes were used as sensors of intestinal lipid absorption from a reconstituted intestinal barrier using Caco-2 and HT29-MTX cells [18]. Based on these different observations and aside from some limitations, the development of an in vitro system combining Caco-2 cells and human primary adipose cells represents an interesting strategy to mimic the intestinal passage of oral products and their potent effects on targeted human adipose tissue.

Furthermore, nutraceuticals and botanicals have been demonstrated, in separate studies, to have significant effects in adipocyte and fat mass modulation. However, most of them were performed on animal in vitro or in vivo models which do not allow a direct comparison of efficacy between them and further demonstrate their limitations, hence weakening their physiological relevance to humans. Indeed, differences between human and animal adipose tissue metabolism are well documented [19, 20]. Moreover, another important bias is the lack of understanding of the mechanisms involved in the local bioavailability substances, as documented by the limited in vivo activity of polyphenols*,* when compared to in vitro studies [21, 22]. Indeed, some polyphenols cannot be absorbed in their native form and require hydrolysis and/or (de-)conjugation to facilitate their penetration through apical transporters. As a result, epithelial cells can metabolize polyphenols back into a glucuronidated form, which can then, be excreted by basolateral transporters [23, 24].

**Fig. 1:**
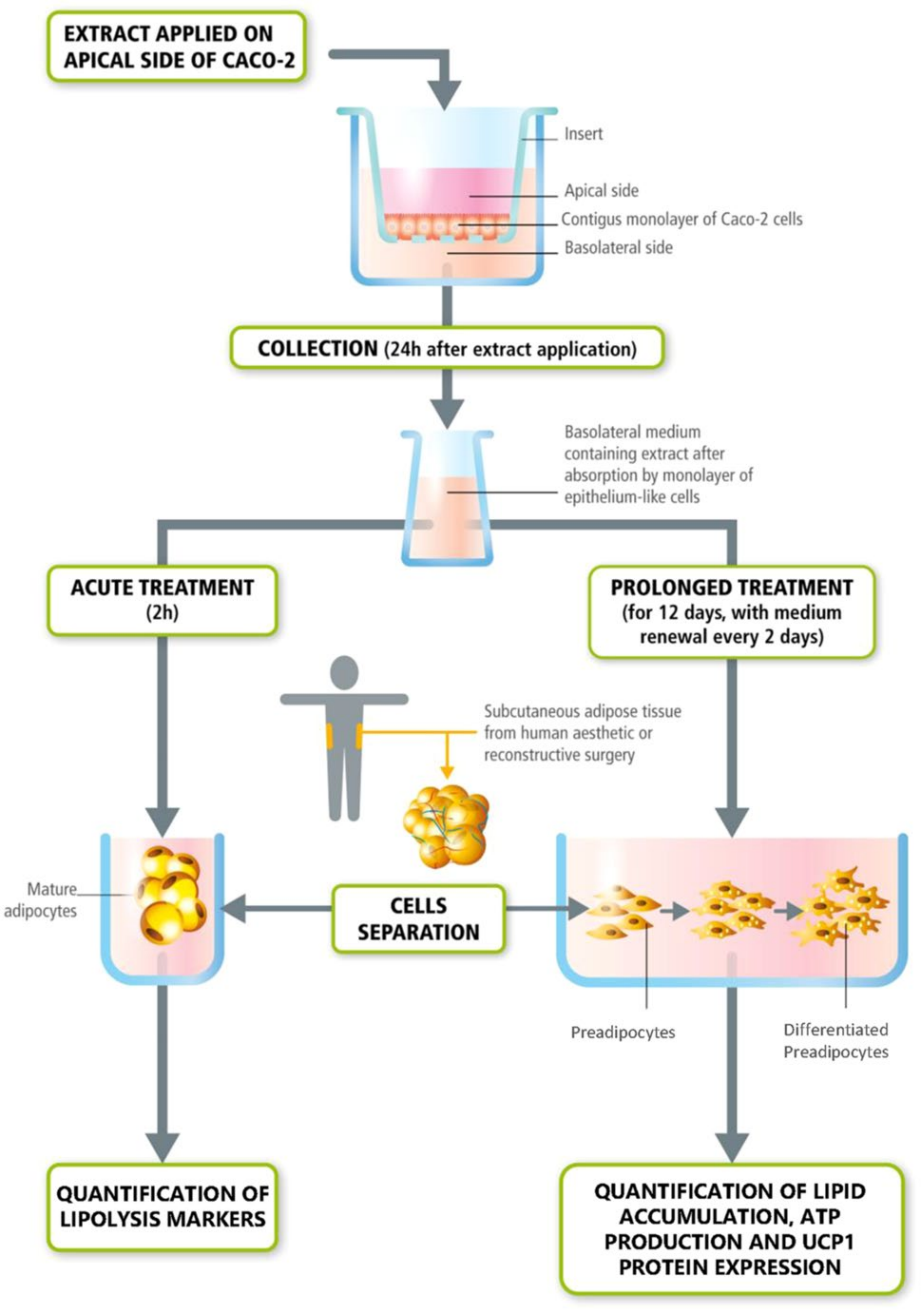
Schematic representation of the in vitro bi-phasic culture system. The first stage consists of aqueous plant extract application on Caco-2 cell monolayer inserts, then the basolateral medium (BM) is recovered after 24 h to mimic in vivo intestinal passage. The second stage consists of collected BM application on human adipose cells in 2 different models: acute treatment (2 h) of mature adipocyte (for lipolysis evaluation throughout NEFA and glycerol release) and prolonged treatment (10–12 days) of pre-adipocytes in pro-adipogenic condition (for lipid accumulation, ATP production and UCP1 protein expression)

To this end, in this study, we set up an in vitro system comprising a bi-phasic culture, combining the Caco-2 cell line monolayer, with primary human adipose cells. Hence, several botanical extracts and their combinations, as compared to coffee extract standard, were used after traversing the Caco-2 monolayer, in characterizing their effects on human adipose cells (Fig. 1).

## Materials and methods

### Botanical extract preparation and posology

All edible botanical ingredients were extracted, dried and made into the powder form prior to use in the trials. Table 1 describes the botanical source, the Latin name, the plant part used, and the process of extraction applied for each selected botanical extract. For each of them, a concentration factor, relative to the botanical reference, was calculated based on the yield of extraction and the hypothetical posology correlation. This initial concentration factor was used for application in cytotoxicity trials on Caco-2 cells. Depending on the cytotoxicity score obtained, final concentrations were defined and used for the conduction of the entire in vitro trial (supplemental results).

**Table 1:** Botanical source, plant part, process of extraction and relative initial/final concentrations tested for cytotoxicity on Caco-2 cells

Code	Latin name	Vernacular name	Plant part	Extraction (Raw material:Solvent)	Initial concentration (g/L)	Final concentration (g/L)
	*Coffea arabica*	Green coffee	Seeds (crushed)	S/L water extraction-1:10	0.72	0.72
A	*Morus alba*	White mulberry	Leaves (dried)	S/L water extraction-1:15	0.72	0.72
B	*Vigna angularis*	Red adzuki	Seeds (whole)	S/L water extraction-1:10	0.36	0.36
C	*Balanites aegyptiaca*	Desert date	Cake (residue after seed pressing)	S/L water extraction-1:10	1.44	0.18
D	*Magnolia officinalis*	Magnolia	Bark (dried. Crushed)	S/L water extraction-1:20	0.72	0.36
E	*Olea europea*	Olive	Fruits (olive mill wastewater)	absorbent resin purification	0.72	0.36
F	*Aframomum melegueta*	Grains of paradise	Seeds (crushed)	S/L water extraction-1:10	0.18	0.18
G	*Cinnamomum aromaticum*	Chinese cinnamon	Bark (crushed)	S/L water extraction-1:10	0.36	0.072
H	*Nelumbo nucifera*	Lotus	Flowers (dried)	S/L water extraction-1:20	0.54	0.54
J	*Mangifera indica*	Mango	Fruit peel (dried crushed)	S/L water extraction-1:10	0.54	0.18
L	*Acacia catechu*	Catechu	Bark (dried. Crushed)	S/L water extraction (0.1 NaOH)-1:10	1.44	0.72
M	*Arctium lappa*	Greater burdock	Seeds (whole)	S/L water extraction-1:10	0.54	0.18
N	*Commiphora mukul*	Guggul	Tree exudate (dried. Crushed)	S/L water extraction-1:10	0.54	0.18
O	*Daucus carota*	Carrot	Seeds (whole)	S/L water extraction-1:10	0.36	0.18
P	*Perilla frutescens*	Shiso	Seeds (whole)	S/L water extraction-1:10	0.18	0.036
Q	*Perilla frutescens*	Shiso	Leaves (dried. Crushed)	S/L water extraction-1:10	0.54	0.18
R	*Cinnamomum verum*	Ceylon cinnamon	Leaves (dried. Crushed)	S/L water extraction-1:10	0.18	0.036
S	*Alpinia galangal*	Greater galangal	Rhizome (dried. Crushed)	S/L water extraction-1:10	0.54	0.36
T	*Salacia reticulata*	Salacia	Roots (dried. Crushed)	S/L water extraction-1:11	0.54	0.36
U	*Syzygium aromaticum*	Clove	Flower buds	S/L water extraction-1:10	0.18	0.072
V	*Rosa canina*	Rose Hip	Fruits (dried. Crushed)	S/L water extraction-1:10	0.72	0.72
W	*Syzygium cumini*	Jambolan	Seeds (crushed)	S/L water extraction-1:10	0.36	0.36
X	*Fragaria* x *ananassa*	Strawberry	Seeds (achens)	S/L hydro-alcoholic (50/50) extraction-1:10	0.18	0.18
Y	*Urtica dioica*	Nettle	Leaves (dried)	S/L water extraction	0.18	0.18
Z	*Jasminum officinalis*	Jasmine (white)	Flowers (whole)	S/L water extraction-1:20	0.72	0.72
Aa	*Helichrysum italicum*	Immortelle	Aerial parts (dried. Crushed)	Water extraction-1:15	0.54	0.54

### Verification of the use of green coffee as a standard plant extract

An extract of green coffee containing caffeine and chlorogenic acid, was used as the reference product, as its properties of modulating adipocyte metabolism, are well documented [25–27].

After verifying the absence of cytotoxic effects (Suppl. Fig.S1), the impact of this extract on pre-adipocyte differentiation was analyzed by assessing lipid accumulation in human pre-adipocytes.

The lipolytic activity of adipocytes was evaluated by measuring the extracellular release of glycerol and non-esterified fatty acids (NEFA) after stimulation with the lipolytic agents Forskolin (1 µm) and Isoproterenol (0.1 µm), or with the basolateral medium from Caco-2 cells in contact or not with green coffee extract. The effect of green coffee on the mitochondrial activity of pre-adipocytes was also carried out to determine the relevance of using the combined Caco-2 with adipose cells, by confirming the caffeine effect of green coffee as a standard, so that other plant extracts could then be tested. As UCP1 is strongly expressed in differentiated tissues as brown/beige adipose tissue, it was quantified in the differentiated pre-adipocytes. The effect of green coffee extract on the UCP1 levels on pre-adipocytes was determined. Finally, mitochondrial ATP synthase activity was also analyzed by quantifying the production of ATP. The condition **BM + green coffee extract** corresponds to basolateral media of caco-2 cells exposed to green coffee extract and is considered as the reference product condition to verify the model.

### Caco-2 culture and differentiation

Caco-2 cells were seeded at a density of 25 × 10^4^ cells on transwells (0.4 µm pore-size polycarbonate membranes) placed in 6-well plates at 37 °C and 5% CO_2_. Apical chambers were filled with 1.5 ml of complete medium (DMEM supplemented with 20% heat inactivated Fetal Bovine Serum (FBS), 1% non-essential amino acids and 1% antibiotics) and the basolateral chamber with 2.5 ml of the same medium. Culture media were changed every day for 21 days to obtain confluent differentiated cell monolayers [14, 28]. Then, the differentiated Caco-2 cells on the apical side were treated with predefined concentrations of the plant extracts (Table 1). After a 24-h incubation period, basolateral media (BM) were collected and frozen at − 80 °C for subsequent experiments on human adipose cells. The condition BM–extract corresponds to basolateral media of caco-2 cells not exposed to botanical extracts and is considered as the control condition to compare the effects of botanical extracts with.

### Plant extract cytotoxicity on Caco-2

Caco-2 cells were seeded in 96-well plates and confluent differentiated cell monolayers were treated with predefined concentrations of the extracts for 48 h. Culture media were collected, and the cytotoxic effect of the extracts was determined by cytotoxic scoring including the activity of the lactate dehydrogenase (LDH, CytoTox-OneTM Fluorescent Assay, G7891, Promega) released, as well as, cell morphology and cell mortality rate after trypan blue staining (15,250,061, Gibco). These scores ranged from 1 (not cytotoxic) to 5 (very highly toxic) and were defined by comparing with the untreated control Caco-2 cells. A botanical extract was considered as cytotoxic if its three scores were superior to or equal to 3 (Suppl. Fig. S2).

### Determination of the cytotoxic concentrations of plant extracts

The non-cytotoxic concentrations of the extracts on Caco-2 cells were determined using cytotoxic scoring, based on lactate dehydrogenase (LDH) release, the morphological aspect of the cells and the rate of cell mortality (Suppl. Fig.S2). The non-cytotoxic concentration of each extract (cytotoxic scoring < 3) for Caco-2 cells, was then used for the 24 h-treatment of differentiated Caco-2 cells. Then, to determine the dilution ratio of basolateral media from Caco-2 culture to apply to adipose cell culture media, 3 dilutions: 1/4, 1/8 and 1/10, were tested for their cytotoxicity on pre-adipocytes, as measured by LDH release and formazan production.

### Cytotoxic effects of the basolateral medium (BM) from Caco-2 cells treated with plant extracts on human pre-adipocytes

Pre-adipocytes were seeded in 96-well plates and were cultured in DMEM-1% FBS containing the basolateral media (BM) from Caco-2 cells treated with the extracts for 24 h, and the media were changed every 2 days throughout the culture period of 6 to 7 days. At the end of the culture period, cytotoxicity was assessed by the measurement of the lactate dehydrogenase (LDH) released in the pre-adipocytes’ culture medium (using CytoTox-OneTM Fluorescent Assay, G7891, Promega), and the number of viable cells was measured using the MTS proliferation assay (CellTiter 96^®^ AQueous One Solution Cell Proliferation Assay, G3580, Promega). The morphological aspect of the cells was monitored all along the culture period.

### Differentiation of human pre-adipocytes

Pre-adipocytes were obtained from overweight (body mass index 25.4 ± 3.1 kg/m^2^) and young (35.5 ± 7.3 years old) female patients undergoing esthetic or reconstructive surgery. Pre-adipocytes were cultured for 24 h in 100 µl of DMEM-10% FBS in 96-well plates. They were then incubated with Caco-2 basolateral media ± tested plant extracts diluted at 1/8 (dilution defined in preliminary study described in Supplemental results) in a pro-adipogenic cocktail containing insulin, glucocorticoid, 3-isobutyl-1-methylxanthine (IBMX), and thiazolinedione [29, 30]. Pre-adipocytes treated with the basal DMEM/F12 medium (undifferentiated) or treated with a PPARγ antagonist (GW9662, 0.1 µm, M6191, Sigma) were used as negative controls of adipocyte differentiation. All conditions were performed in triplicate. The medium was changed every 2 days for up to 10 to 12 days.

### Quantification of lipid accumulation and of UCP1 protein expression

After 10–12 days of culture, pre-adipocytes were fixed with 4% paraformaldehyde then washed with PBS containing 0.1 M glycine. Following permeabilization by PBS containing 3% BSA and 0.1% triton X100, Uncoupling Protein 1 (antibody against UCP1, U6382, Sigma) staining was performed at 4 °C, overnight. Bodipy (D3922, Molecular Probes), and DAPI (4',6-Diamidino-2-Phenylindole, Dihydrochloride) (D1306, Molecular Probes) staining were performed at room temperature to stain for lipid droplets and nuclei, respectively.

Quantifications of lipid accumulation and UCP1 protein expression were collected from image acquisition and processing was performed according to the procedure specifically developed as described in the supplemental materials and methods.

### ATP production assay

ATP was measured in pre-adipocytes cultured for 10–12 days in adipogenic or non-adipogenic differentiation medium, using a luminescent ATP Detection Reagent (Mitochondrial ToxGloAssay, G8000, Promega). The mitochondrial toxin Rotenone (400 nM, R8875, Sigma) was used as a negative control. DAPI fluorescent staining was performed to evaluate cell number and to normalize the luminescence signal.

### Adipocyte culture and measurement of lipolytic activity

Adipocytes were incubated in Krebs Ringer Bicarbonate (KRB) buffer supplemented or not with basolateral medium from Caco-2 cells treated with either predetermined concentrations of the extracts, at a dilution of 1/8, or the pro-lipolytic reference products (Forskolin and Isoproterenol), at 37 °C for 2 h. Forskolin (1 µm, F6886-Sigma) and isoproterenol (0.1 µm, I6504-Sigma) were used as activators of lipolysis. Cell culture media were then collected and frozen at − 20 °C. The lipolytic activity of human adipocytes was assessed by the measurement of glycerol (Glycerol assay, GY105, Randox), and non-esterified fatty acid release (NEFA-HR2 R1 Set, 434-91795 et NEFA-HR2 R2 Set, 436-91995, Wako) in the adipocyte culture medium. Adipocytes were collected for DNA quantification (Quant-iT™ PicoGreen™ dsDNA Assay Kit, P7589, Invitrogen) to normalize glycerol and NEFA concentrations.

### Statistical analysis

Results are expressed as percentages of respective controls. Statistical analyses were performed using the Prism software (GraphPad Software). We used triplicate assays for each experiment per donor, resulting in a total of nine values for the three donors. Comparisons between the treatment and control conditions (no extract) were analyzed by the Friedman test, to compare repeated data for dependent samples, followed by the Wilcoxon test, to compare paired samples two by two. Differences were considered as statistically significant when *p* ≤ 0.05 (*p ≤ 0.05; **p ≤ 0.01; ***p ≤ 0.001; ****p ≤ 0.0001).

## Results

### Verification of the culture system combining human Caco-2 cells and adipose cells using green coffee extract

#### The effect of green coffee extract on pre-adipocyte differentiation

**Fig. 2:**
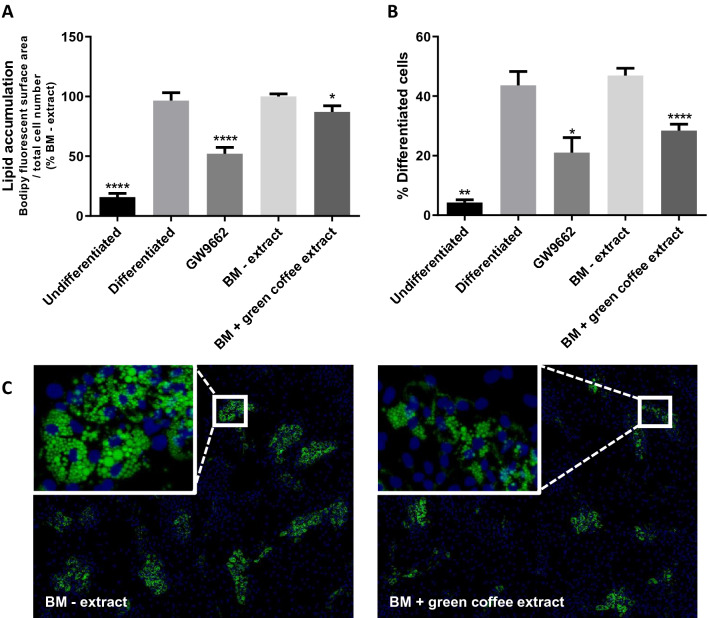
Lipid accumulation in human pre-adipocytes is decreased with Caco-2 basolateral medium + green coffee extract (BM + green coffee extract) compared to control Caco-2 basolateral medium (BM – extract). **A** Quantification of lipid accumulation after BODIPY staining (fluorescent surface area relative to total cell number), *n* = 9. **B** Percentage of differentiated cells relative to total cell number, *n* = 3. *p < 0.05; **p < 0.01; ****p < 0.0001. **C** Representative immunofluorescent microphotographs of lipid droplets (BODIPY in green) and nuclei (DAPI in blue) staining. Undifferentiated: pre-adipocytes in basal culture medium; Differentiated: pre-adipocytes in pro-adipogenic culture medium; GW9662: pre-adipocytes in pro-adipogenic culture medium with PPARγ antagonist, GW9662 (1 µm); BM – extract: pre-adipocytes in pro-adipogenic culture medium with basolateral media of caco-2 cells not exposed to botanical extracts; BM + green coffee extract: pre-adipocytes in pro-adipogenic culture medium with basolateral media of caco-2 cells exposed to green coffee extract

After verifying the absence of cytotoxic effects (Suppl. Fig.S1), the impact of this extract on pre-adipocyte differentiation was analyzed by assessing lipid accumulation in human pre-adipocytes. GW9662, an antagonist of PPARγ, diminished lipid accumulation by about 50% compared to the BM–extract (Fig. 2A). Green coffee extract (BM + green coffee extract) decreased lipid accumulation by about 13% (Fig. 2A, C) compared to the BM–extract. The percentage of differentiated cells was also reduced by about 40% by green coffee extract (Fig. 2B, C).

#### The effect of green coffee extract on the lipolytic activity of adipocytes

**Fig. 3:**
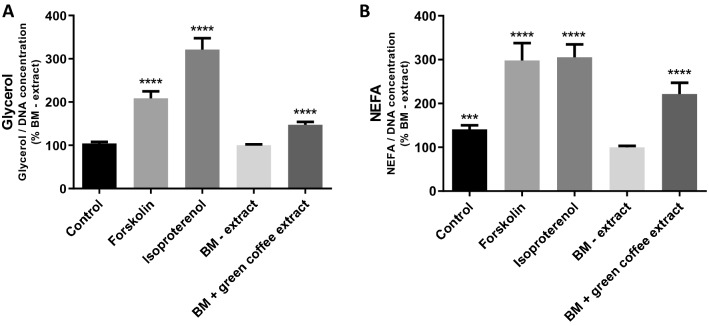
Lipolytic activity of human adipocytes is increased with Caco-2 basolateral medium + green coffee extract (BM + green coffee extract) compared to control Caco-2 basolateral medium (BM – extract). **A** Quantification of glycerol release (concentration relative to DNA quantification), *n* = 9. **B** Quantification of NEFA release (concentration relative to DNA quantification), *n* = 9. ***p < 0.001; ****p < 0.0001. Control: mature adipocytes in basal culture medium; Forskolin: mature adipocytes in specific adipocyte culture medium with Forskolin (1 µm); Isoproterenol: mature adipocytes in specific adipocyte culture medium with Isoproterenol (0.1 µm); BM – extract: mature adipocytes in specific adipocyte culture medium with basolateral media of caco-2 cells not exposed to botanical extracts; BM + green coffee extract: mature adipocytes in specific adipocyte culture medium with basolateral media of caco-2 cells exposed to green coffee extract

Forskolin and isoproterenol increased glycerol release by about 100% and 220%, respectively, and increased NEFA release by about 200%. Green coffee extract (BM + green coffee extract) significantly increased the glycerol (Fig. 3A) and NEFA (Fig. 3B) release by 50% and 120%, respectively, compared to the BM–extract.

#### The effect of green coffee extract on mitochondrial activity of pre-adipocytes

**Fig. 4:**
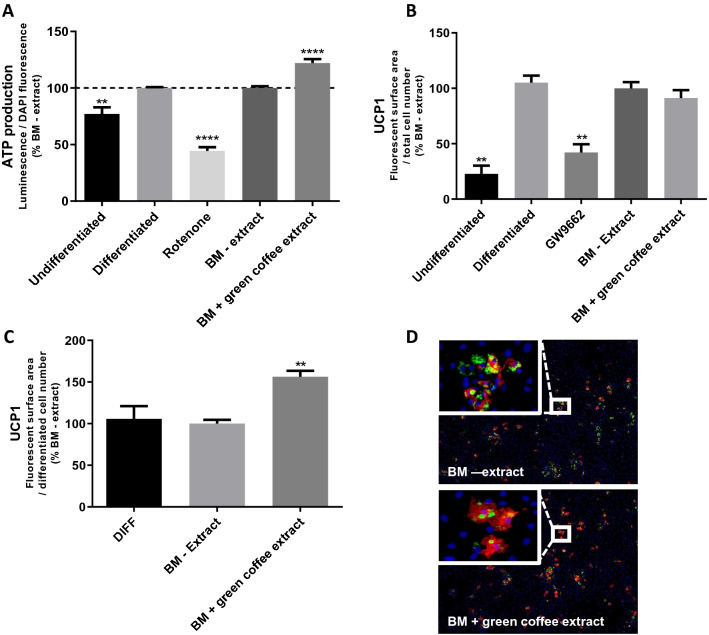
Energetic metabolism of human pre-adipocytes is increased with Caco-2 basolateral medium + green coffee extract (BM + green coffee extract) compared to control Caco-2 basolateral medium (BM – extract). **A** Quantification of ATP production (luminescence relative to DAPI fluorescence), *n* = 4. **B** Quantification of UCP1 protein expression after immunostaining in total cells (fluorescent surface area relative to total cell number), *n* = 3. **C** Quantification of UCP1 protein expression after immunostaining in differentiated pre-adipocytes (fluorescent surface area relative to differentiated cell number), *n* = 3. **p < 0.01; ****p < 0.0001. **D** Representative micro-photographs of UCP1 (red), lipid accumulation (BODIPY in green) and nuclei (DAPI in blue) staining. Undifferentiated: pre-adipocytes in basal culture medium; Differentiated or DIFF: pre-adipocytes in pro-adipogenic culture medium; GW9662: pre-adipocytes in pro-adipogenic culture medium with PPARγ antagonist, GW9662 (1 µm); Rotenone: pre-adipocytes in pro-adipogenic culture medium with mitochondrial toxin, Rotenone (400 nM); BM – extract: pre-adipocytes in pro-adipogenic culture medium with basolateral media of caco-2 cells not exposed to botanical extracts; BM + green coffee extract: pre-adipocytes in pro-adipogenic culture medium with basolateral media of caco-2 cells exposed to green coffee extract

Mitochondrial ATP synthase activity was analyzed by quantifying the production of ATP. As shown in Fig. 4A, treatment with the negative control rotenone decreased ATP production by 55% in differentiated pre-adipocytes. Green coffee extract (BM + green coffee extract) increased the ATP production significantly (+ 20%) in differentiated pre-adipocytes when compared to BM – extract (Fig. 4A). In addition, when normalized to the total cell number, the adipogenesis inhibitor GW9662 [31], significantly inhibited UCP1 protein (-60%) expression when compared to the BM–extract (Fig. 4B), but the green coffee extract (BM + green coffee extract) did not have any effect on UCP1 levels when compared to BM–extract. However, when normalized only to the number of differentiated cells, UCP1 protein expression was significantly increased by 55% in cells differentiated in the presence of green coffee extract (BM + green coffee extract) compared to BM – extract (Fig. 4C, D).

### The adipocyte/Caco-2 culture system is suitable for the evaluation of the effects of 25 plant extracts on adipose cell biology

#### Cytotoxicity of plant extracts

Three dilutions, 1/4, 1/8 and 1/10, of Caco-2 basolateral media were tested for their toxicity on pre-adipocytes by the eventual release of LDH and formazan production. Dilutions of 1/8 and 1/10 were considered not to be cytotoxic for pre-adipocytes and hence the lowest dilution of 1/8, was used in the subsequent analyses, so as not to dilute the extracts too much (Suppl. Fig.S3).

#### Effects of the 25 plant extracts on pre-adipocyte differentiation and adipocyte lipolysis [32]

**Fig. 5:**
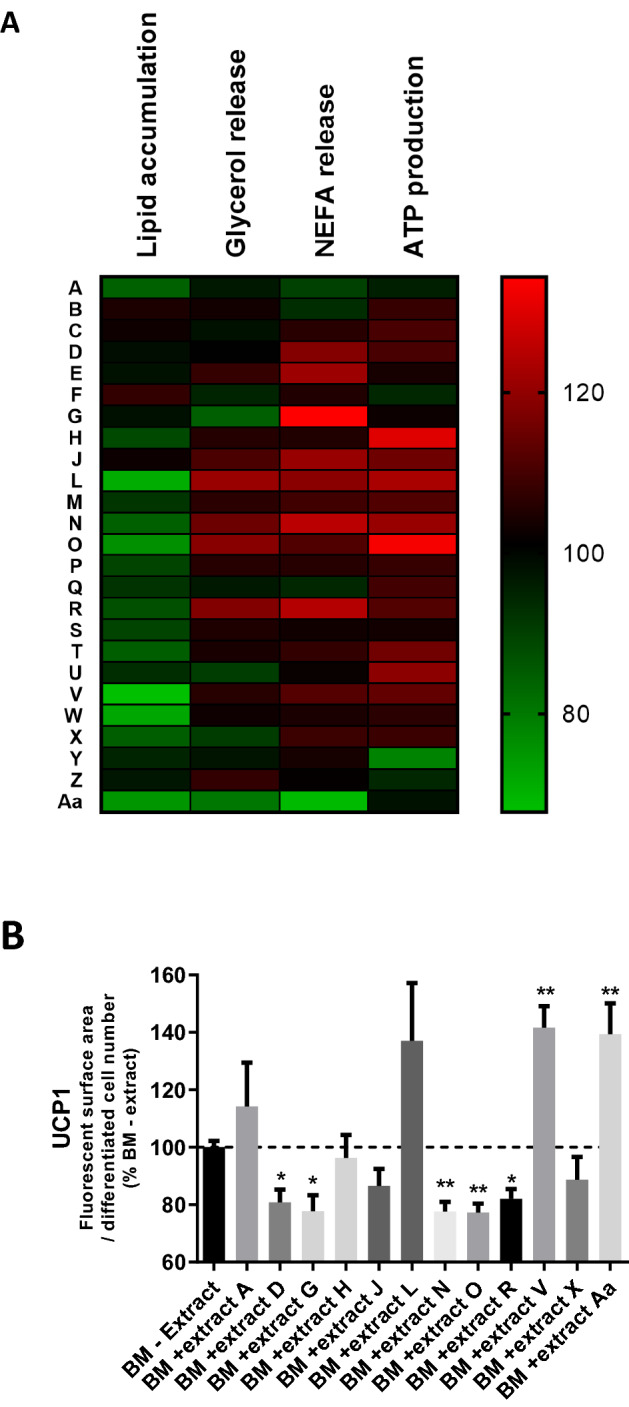
Biological effects of plant extracts on human pre-adipocytes and mature adipocytes. **A** Heat-map illustration displaying the effects of plant extracts on lipid accumulation and ATP production in human pre-adipocytes and on glycerol and NEFA release in human mature adipocytes, *n* = 3 to 6. Each row represents a plant extract and columns represent biological parameters. The red region corresponds to stimulatory effects and green region corresponds to inhibitory effects. **B** Quantification of UCP1 protein expression after immunostaining in differentiated pre-adipocytes (fluorescent surface area relative to differentiated cell number), *n* = 3. BM – extract: pre-adipocytes in pro-adipogenic culture medium with basolateral media of caco-2 cells not exposed to botanical extracts; BM + extract LETTER (A to Aa): pre-adipocytes in pro-adipogenic culture medium with basolateral media of caco-2 cells exposed to plant extracts described in Table 1

The cytotoxicity of the BM from Caco-2 cells incubated with the 25 extracts (BM + extract latter) on pre-adipocytes was tested using the LDH assay. BM ± products were not cytotoxic for pre-adipocytes and did not affect their viability (Suppl. Fig.S4). Upon evaluating the effects of the extracts on pre-adipocyte differentiation, in comparison with BM–extract, two extracts, A and J, significantly increased lipid accumulation by about 15%, while six extracts, L, O, P, S, T and W, significantly decreased it by 15%, 20%, 10%, 10%,15% and 28% respectively (Fig. 5A and Suppl. Fig.S5). The others had no significant effect on lipid accumulation. Finally, the two markers of lipolysis, glycerol and NEFA release, were analyzed. Extracts J, L, O and R, increased glycerol release by mature adipocytes significantly, by about 10%, 20%, 18% and 18%, respectively, while two extracts, G and Aa decreased it by 15% and 20%, respectively (Fig. 5A and Suppl. Fig.S5). The release of NEFA was increased by L and N extracts by about 20% and 25% and a similar trend was observed with extract R, while extract Aa decreased it by 30% (Fig. 5A and Suppl. Fig.S5).

#### Effects of the 25 plant extracts on mitochondrial activity of differentiated pre-adipocytes

The production of ATP by differentiated pre-adipocytes after 10–12 days of culture in pro-adipogenic medium, increased with two extracts, H and X, by + 30% and + 10% respectively when compared to BM–extract. Three additional extracts J, L and O, showed a similar tendency to increase in this parameter. Only one extract, Y, decreased it significantly by 20%, while the other extracts did not have any significant effect (Fig. 5A and Suppl. Fig.S5).

#### UCP1 protein expression in differentiated pre-adipocytes after plant extract treatment

Following the previous results of lipid accumulation, ATP production and lipolysis, twelve extracts (A, D, G, H, J, L, N, O, R, V, X and Aa) were assessed for their effects on the protein expression of UCP1 in differentiated pre-adipocytes in comparison with the condition BM–extract. The expression of this protein relative to the number of differentiated cells increased significantly after treatment with V and Aa extracts by about 40% and tended to increase with extract L (Fig. 5B and Suppl. Fig.S5). The extracts D, G, N, O and R significantly decreased UCP1 expression by about 20%.

To sum up (Suppl. Fig.S5), only a few of the extracts exhibited an overall adipocyte modulation similar to green coffee extract (H, L, N, O, R, V and Aa), even though they were tested at a much lower concentration (especially N, O and R). Only V and Aa increased the expression of the UCP1 protein in differentiated pre-adipocytes, and in a comparable manner to green coffee. As these extracts could have different specific modulation of adipocyte activities, a combination of them was tested to assess if a synergistic activity could be achieved.

### Effects of 6 combinations of plant extracts on pre-adipocytes after 24 h of treatment on Caco-2 cells

In this part of the study, we aimed to evaluate the effects of plant extract combinations (Table 2). In total, six combinations were tested. These extracts were selected according to their potential properties as “fat mobilizers” (increasing lipolysis), “fat burners” (increasing energetic metabolism) or “fat inhibitors” (decreasing lipid accumulation).

**Table 2:** Botanical source of seven extracts used for combination tests

Combination	Code	Vernacular name
1	L	Catechu
	N	Guggul
2	L	Catechu
	Aa	Immortelle
3	H	Lotus
	Aa	Immortelle
4	O	Carrot
	V	Rose Hip
5	L	Catechu
	V	Rose Hip
6	O	Carrot
	R	Ceylon cinnamon

#### Effects of plant extracts combinations on lipid accumulation in pre-adipocytes and on adipocyte lipolysis

**Fig. 6:**
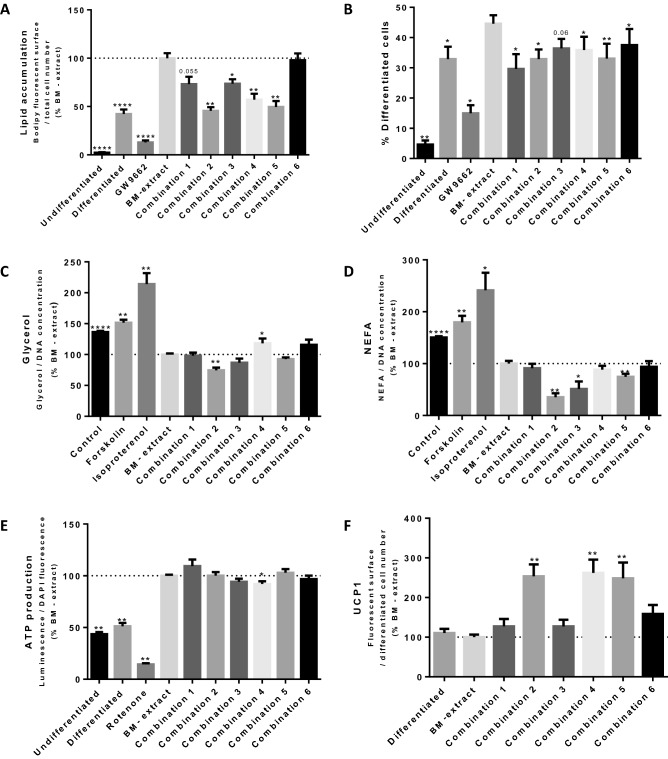
Effects of combinations of plant extracts on adipose cells. **A** Quantification of lipid accumulation in pre-adipocytes (fluorescent surface area relative to total cell number), *n* = 3. **B** Percentage of differentiated cells relative to total cell number, *n* = 3. *p ≤ 0.05; **p ≤ 0.01; ***p ≤ 0.001; ****p ≤ 0.0001. Undifferentiated: pre-adipocytes in basal culture medium; Differentiated: pre-adipocytes in pro-adipogenic culture medium; GW9662: pre-adipocytes in pro-adipogenic culture medium with PPARγ antagonist, GW9662 (1 µm); BM – extract: pre-adipocytes in pro-adipogenic culture medium with basolateral media of caco-2 cells not exposed to botanical extracts; BM + Combination 1 to 6: pre-adipocytes in pro-adipogenic culture medium with combination of basolateral media of caco-2 cells exposed to plant extracts described in Table 3. **C** Quantification of Glycerol release in mature adipocytes (concentration relative to DNA quantification), *n* = 3. **D** Quantification of NEFA release in mature adipocytes (concentration relative to DNA quantification), *n* = 3. *p ≤ 0.05; **p ≤ 0.01; ***p ≤ 0.001; ****p ≤ 0.0001. Control: mature adipocytes in basal culture medium; Forskolin: mature adipocytes in specific adipocyte culture medium with Forskolin (1 µm); Isoproterenol: mature adipocytes in specific adipocyte culture medium with Isoproterenol (0.1 µm); BM – extract: mature adipocytes in specific adipocyte culture medium with basolateral media of caco-2 cells not exposed to botanical extracts. BM + Combination 1 to 6: mature adipocytes in specific adipocyte culture medium with combination of basolateral media of caco-2 cells exposed plant extracts described in Table 3. **E** Quantification of ATP production in pre-adipocytes (luminescence relative to DAPI fluorescence), *n* = 3. *p ≤ 0.05; **p ≤ 0.01; ***p ≤ 0.001; ****p ≤ 0.0001. Undifferentiated: pre-adipocytes in basal culture medium; Differentiated or DIFF: pre-adipocytes in pro-adipogenic culture medium; Rotenone: pre-adipocytes in pro-adipogenic culture medium with mitochondrial toxin, Rotenone (400 nM); BM – extract: pre-adipocytes in pro-adipogenic culture medium with basolateral media of caco-2 cells not exposed to botanical extracts; BM + Combination 1 to 6: pre-adipocytes in pro-adipogenic culture medium with combination of basolateral media of caco-2 cells exposed to plant extracts described in Table 3. **F** Quantification of UCP1 protein expression after immunostaining in differentiated pre-adipocytes (fluorescent surface area relative to differentiated cell number), *n* = 3. *p ≤ 0.05; **p ≤ 0.01; ***p ≤ 0.001; ****p ≤ 0.0001. Differentiated: preadipocytes in proadipogenic culture medium; BM – extract: preadipocytes in proadipogenic culture medium with basolateral media of caco-2 cells not exposed to botanical extracts; BM + Combination 1 to 6: preadipocytes in proadipogenic culture medium with combination of basolateral media of caco-2 cells exposed to plant extracts described in Table 3

After assessing the absence of cytotoxicity of the extract combinations (Suppl. Fig.S6), pre-adipocyte differentiation was evaluated by quantifying their lipid accumulation. As expected, the PPARγ antagonist GW9662 diminished lipid accumulation significantly by 90% when compared to BM – extract. Interestingly, four combinations, 2, 3, 4 and 5 significantly decreased lipid accumulation in pre-adipocytes by 55%, 25%, 40% and 50%, respectively, while combination 1 tended to decrease it, and combination 6 did not present any effects (Fig. 6A). The percentage of differentiated cells was also reduced by all the combinations (Fig. 6B). As for forskolin or isoproterenol treatment, only combination 4 showed potent lipolytic properties as demonstrated by the significant increase in glycerol release (+ 20%), whereas no increase of NEFA was measured (Fig. 6C, D). Combination 2 significantly reduced both the glycerol and NEFA release and to a lower extent combination 3, which showed only a minor lipolytic effect (Fig. 6C, D).

#### Mitochondrial activity and UCP1 expression after treatment of pre-adipocytes with combinations of plant extracts

Evaluation of ATP production showed that combination 4 exhibited a significant inhibitory effect (-10%), while the other combinations did not have any effect (Fig. 6E). This observation correlated with the significant increase of UCP1 expression (+ 150%) with combination 4 (Fig. 6F). Similar increase in UCP1 expression was observed with combinations 2 and 5 without affecting ATP production (Fig. 6E, F).

Taken together (Table 3), these data indicate that combinations 2, 3, 4 and 5 significantly inhibited lipid accumulation in differentiated pre-adipocytes, with the highest efficacy achieved with combinations 2 (*Acacia catechu* and Immortelle-45.5%) and 5 (*Acacia catechu* and Rose hip-49.6%). A moderate effect on lipolysis was observed with combination 4 (and contrary to combinations 2 and 5) only, while for all combinations, modulations of ATP were not significant or were very weak. Finally, combinations 2, 4 (*Daucus carota* and Rose hip) and 5 exhibited the highest significant effects seen in the increase of UCP1 protein expression in differentiated pre-adipocytes (253.4%, 262.1% and 248.5% respectively).

**Table 3:** Summary table of biological effects of plant extract combinations on adipose cells

Combination	Vernacular name	Code	Lipid accumulation	Glycerol release	NEFA release	ATP Production	UCP1 expression/Total cell number	UCP1 expression/Differentiated cells
1	Catechu	L	73.21 (7.64)	98.26 (4.79)	91.20 (8.40)	109.29 (6.40)	100.50 (10.27)	127.22 (18.64)
	Guggul	N						
2	Catechu	L	45.52** (3.82)	74.38** (4.45)	35.38** (7.51)	100.20 (3.35)	146.69* (13.67)	253.42** (30.17)
	Immortelle	Aa						
3	Lotus	H	73.65* (4.57)	86.87 (6.64)	51.13* (14.53)	94.20 (3.04)	110.38 (7.76)	127.12 (16.87)
	Immortelle	Aa						
4	Carrot	O	57.05** (6.17)	118.24* (7.71)	88.38 (7.49)	92.11* (2.60)	168.81* (14.52)	262.13** (33.54)
	Rose Hip	V						
5	Catechu	L	49.57** (6.11)	92.66 (2.75)	74.74** (5.56)	102.96 (3.51)	135.54* (12.72)	248.51** (39.69)
	Rose Hip	V						
6	Carrot	O	97.90 (7.06)	115.72 (8.29)	93.89 (11.07)	96.83 (3.42)	157.76* (19.51)	158.55 (22.67)
	Ceylon cinnamon	R						

Results were expressed in percentage relative to the control

Mean ± SEM (standard error of the mean) of *n* = 3

^*^p < 0.05

^**^p < 0.01

## Discussion

The accumulation of fat leads to obesity and hence to potentially serious health problems, such as cardiovascular disease or diabetes. Much time and effort have been put in finding solutions to either minimize fat accumulation in the first place, or to reduce stored fat through increased metabolism or changing adipose composition. In recent years, many patients and even the general population resort to plant derived products to control or reduce their fat buildup. However, the effectiveness of these substances on the adipose tissue has not always been looked at or confirmed. In this study, we aimed to bring together an in vitro model of the absorption of plant extracts by the intestinal barrier with their eventual effect on human adipose tissue and hence to find the most active extracts to this effect.

Hence, this bi-phasic in vitro trial compared botanical extract potencies intended for oral use for their biological activities on lipid accumulation, adipocyte lipolysis or energy metabolism using an in vitro model combining intestinal Caco-2 cells and human adipose cells. First, this culture system was verified by establishing its dose-dependent response using a standardized well-known botanical ingredient, green coffee. Second, a variety of botanical extracts were differentiated from each other by determining the differences in the way they regulate adipocyte physiology. Finally, the most effective extracts, and their combinations, were used to characterize and to further investigate their actions on adipose cells.

The first part of the model was represented by a monolayer of Caco-2 cells to mimic intestinal passage, as in, the absorption of substances from the internal-side of the epithelial membrane, partial metabolization of the substances by the epithelial cells, and the secretion of the metabolites as well as digestive enzymes into the external-milieu of the epithelial cell. Consequently, in this way, the digestion and release of plant extracts were at least partially considered in this model prior to further in vitro investigation on adipose cells.

To start with, all plant products were extracted with a standard aqueous recipe to recover soluble compounds which would then be released into the GI tract in accordance with dietary botanicals intended for oral use (pills or capsules). Previous caco-2 trials have successfully shown that Caco-2 cells absorb hydrophilic compounds, in much the same way as in vivo intestinal absorption [19, 20, 40], despite some limitations in the mechanisms of bioavailability, such as tissue distribution specificity and interaction with other active compounds.

The second part of the model consisted of the quantification of the biological effects on human adipose cells of plants extract after Caco-2 barrier passage.

To verify the functionality and the biological relevance of this bi-phasic system for our purposes, different doses of a green coffee extract with standardized caffeine and chlorogenic acid concentrations, which are known for their in vivo lipolytic actions in adipose tissue [33–36], were applied to Caco-2 cells. Each of the twenty-five plant extracts was then screened on the same biological parameters and compared with the green coffee extract. Out of these, only seven plant extracts were retained due to their inhibitory effects on lipid accumulation or thermogenic effects through stimulated UCP1 expression or pro-lipolytic activities or which presented two or three of these effects simultaneously: *Acacia catechu* (bark), Immortelle, Rose hip (fruit), *Daucus carota* (seeds), Ceylon Cinnamon, Lotus (flower) and Guggul (exudate).

*Acacia catechu* has previously been described as having anti-oxidant, anti-inflammatory and chemoprotective properties [37, 38] whereas Immortelle is known as an antimicrobial and anti-inflammatory agent [39]. For the first time, our study demonstrates that *Acacia catechu* and Immortelle can modulate adipose cell biology in stimulating lipolysis and mitochondrial activity, respectively. Moreover, they both similarly reduced lipid accumulation in human pre-adipocytes. Interestingly, together, they presented better bio-effects than when tested separately, particularly related to UCP1 expression (+ 153.4% compared to the control condition).

Rose Hip is commonly used as a vitamin C supplement and is known for its antioxidant and anti-inflammatory properties. Preclinical studies have shown that Rose hip prevents body weight gain and visceral fat mass accumulation in non-obese mice [40] and inhibits lipid accumulation in a transformed cell line derived from mouse 3T3-L1 fibroblasts [41]. Furthermore, a clinical study performed on 32 overweight subjects showed that Rose Hip supplements reduced abdominal total fat, visceral fat and body weight [42]. When tested individually, our study revealed that Rose Hip had the best effect in reducing lipid accumulation (− 33% compared to the control condition) and UCP-1 expression in human pre-adipocytes (+ 40% compared to the control condition), which could explain the clinical data described above. When associated with *Acacia catechu*, a significant decrease in lipid accumulation and a stronger induction of UCP1 protein expression were observed suggesting synergistic effects of these two extracts, (+ 148.5% compared to the control condition).

Furthermore, several studies, mainly one in vivo study performed in rats fed a high-fat diet reported that supplementation with carrots led to a decrease in fat mass and weight gain [43–45]. However, the present study showed that although *Daucus carota* seeds activated adipocyte lipolysis and inhibited lipid accumulation in pre-adipocytes, they failed to induce energy metabolism and thermogenesis. Nevertheless, its association with Rose Hip produced the best bio-effect, including stimulated lipolysis, reduced lipid accumulation, and synergistic thermogenic activity (+ 162.1% UCP1 expression compared to the control condition).

Ceylon Cinnamon is considered able to mimic the effects of insulin while promoting 3T3-L1 pre-adipocyte differentiation [46] and upregulating the expression of UCP-1 [47]. However, our study shows that Ceylon Cinnamon alone, induced lipolysis in mature adipocytes and diminished lipid accumulation without affecting the energy metabolism of pre-adipocytes.

Of the seven individual extracts selected, lotus was the only one that has previously been shown to exhibit inhibitory effects on lipid accumulation and on the expression of adipogenic transcription factors in human pre-adipocytes [48–51]. However, we did not find any effect on lipolytic activity, but could confirm the anti-adipogenic effect of the Lotus flower extract and its stimulatory effect on energy metabolism. Furthermore, its combination with Immortelle did not have any added biological interest on lipid accumulation in pre-adipocytes.

Finally, Guggul has already been studied for its anti-adipogenic properties due to its main component, Guggulsterone, which has been shown to inhibit adipogenesis, to induce apoptosis and to stimulate lipolysis in 3T3-L1 cells [52, 53]. In our model, its lipolytic activity in mature human adipocytes and its inhibitory effect on lipid accumulation in human pre-adipocytes, were confirmed. In addition, a novel potential adipose thermogenic activity of this extract was also identified. The combination of Guggul with *Acacia catechu* improved its inhibitory effect on lipid accumulation and interestingly, demonstrated its capacity of inducing mitochondrial activity. However, complementary investigations should be considered to further support the actions of these botanical extract, such as clinical studies in obese subjects. Furthermore, as hypertrophied adipocytes produce pro-inflammatory cytokines and chemokines [29, 54], contributing to the chronic inflammation associated with obesity, it would also be interesting to assess the potential of these extracts in modulating chronic inflammation of adipose tissue in vivo.

In conclusion, we have tested a co-culture model, combining human intestinal and adipose cells which is also taken into account the intestinal passage of oral plant extracts. In addition, this model was primarily verified using, as a standard, green coffee extract, due to its caffeine and chlorogenic acid concentrations.

As a proof of concept, botanical extracts were categorized and the best were chosen according to their different activities in modulating human adipose tissue biology, such as lipolysis, lipid accumulation, energetic homeostasis and thermogenesis activation by UCP1 protein expression. In particular, certain paired combinations of *Catechu*, Immortelle, Carrot and Rose hip showed higher inhibition of lipid accumulation and exceptional activation of thermogenic potency when compared to each the extracts separately (including green coffee), and to other combinations tested. However, further robust in vivo trials would be necessary to validate the prospective interest of these compounds for application on humans.

## Supplementary Information

Below is the link to the electronic supplementary material.

Supplementary file1 (PPTX 2246 KB)
