# Supplementary material for: Screening for anti-adipogenic, pro-lipolytic and thermogenic plant extracts by models associating intestinal epithelial cells with human adipose cells
Source: Eur J Nutr. 2022 Jan 29;61(4):2201–15. doi: 10.1007/s00394-021-02794-8 (PMC9106611; doi:10.1007/s00394-021-02794-8)
Supplement: Supplementary file 1 — Supplementary file1 (PPTX 2246 KB) [file 394_2021_2794_MOESM1_ESM.pptx]

## Slide 1
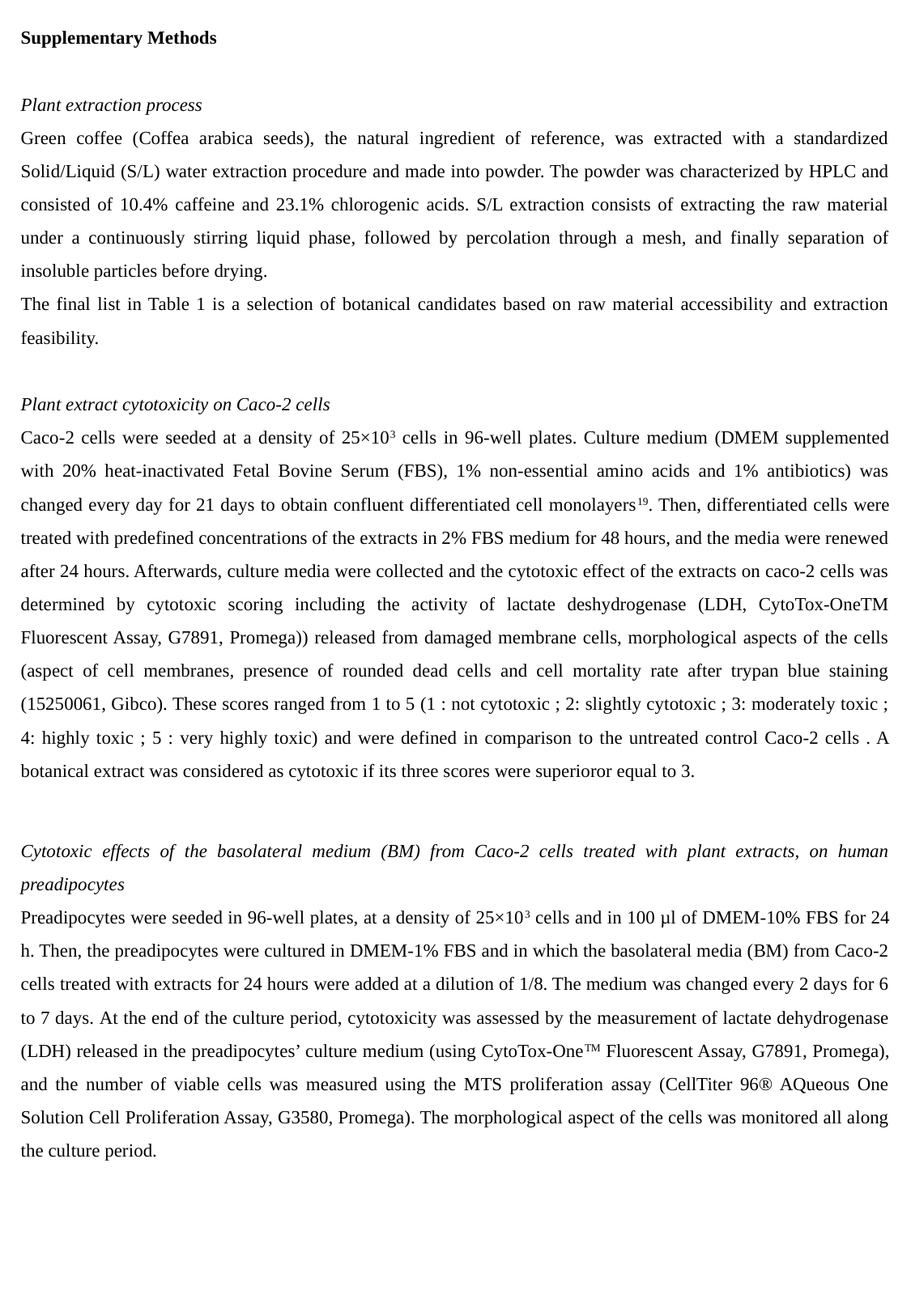

Supplementary Methods
Plant extraction process
Green coffee (Coffea arabica seeds), the natural ingredient of reference, was extracted with a standardized Solid/Liquid (S/L) water extraction procedure and made into powder. The powder was characterized by HPLC and consisted of 10.4% caffeine and 23.1% chlorogenic acids. S/L extraction consists of extracting the raw material under a continuously stirring liquid phase, followed by percolation through a mesh, and finally separation of insoluble particles before drying.
The final list in Table 1 is a selection of botanical candidates based on raw material accessibility and extraction feasibility.
Plant extract cytotoxicity on Caco-2 cells
Caco-2 cells were seeded at a density of 25×103 cells in 96-well plates. Culture medium (DMEM supplemented with 20% heat-inactivated Fetal Bovine Serum (FBS), 1% non-essential amino acids and 1% antibiotics) was changed every day for 21 days to obtain confluent differentiated cell monolayers19. Then, differentiated cells were treated with predefined concentrations of the extracts in 2% FBS medium for 48 hours, and the media were renewed after 24 hours. Afterwards, culture media were collected and the cytotoxic effect of the extracts on caco-2 cells was determined by cytotoxic scoring including the activity of lactate deshydrogenase (LDH, CytoTox-OneTM Fluorescent Assay, G7891, Promega)) released from damaged membrane cells, morphological aspects of the cells (aspect of cell membranes, presence of rounded dead cells and cell mortality rate after trypan blue staining (15250061, Gibco). These scores ranged from 1 to 5 (1 : not cytotoxic ; 2: slightly cytotoxic ; 3: moderately toxic ; 4: highly toxic ; 5 : very highly toxic) and were defined in comparison to the untreated control Caco-2 cells . A botanical extract was considered as cytotoxic if its three scores were superioror equal to 3.
Cytotoxic effects of the basolateral medium (BM) from Caco-2 cells treated with plant extracts, on human preadipocytes
Preadipocytes were seeded in 96-well plates, at a density of 25×103 cells and in 100 µl of DMEM-10% FBS for 24 h. Then, the preadipocytes were cultured in DMEM-1% FBS and in which the basolateral media (BM) from Caco-2 cells treated with extracts for 24 hours were added at a dilution of 1/8. The medium was changed every 2 days for 6 to 7 days. At the end of the culture period, cytotoxicity was assessed by the measurement of lactate dehydrogenase (LDH) released in the preadipocytes’ culture medium (using CytoTox-OneTM Fluorescent Assay, G7891, Promega), and the number of viable cells was measured using the MTS proliferation assay (CellTiter 96® AQueous One Solution Cell Proliferation Assay, G3580, Promega). The morphological aspect of the cells was monitored all along the culture period.

## Slide 2
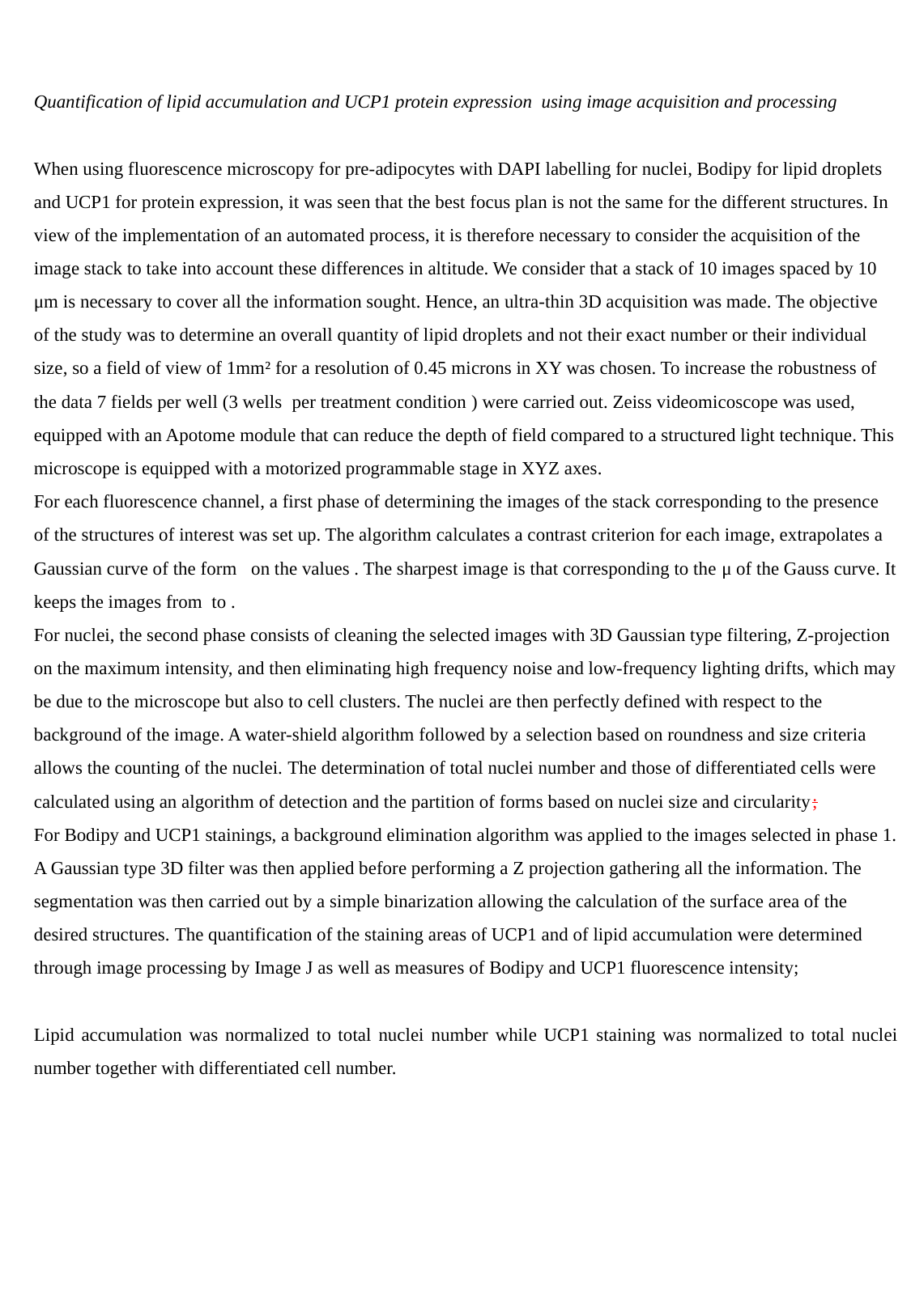

## Slide 3
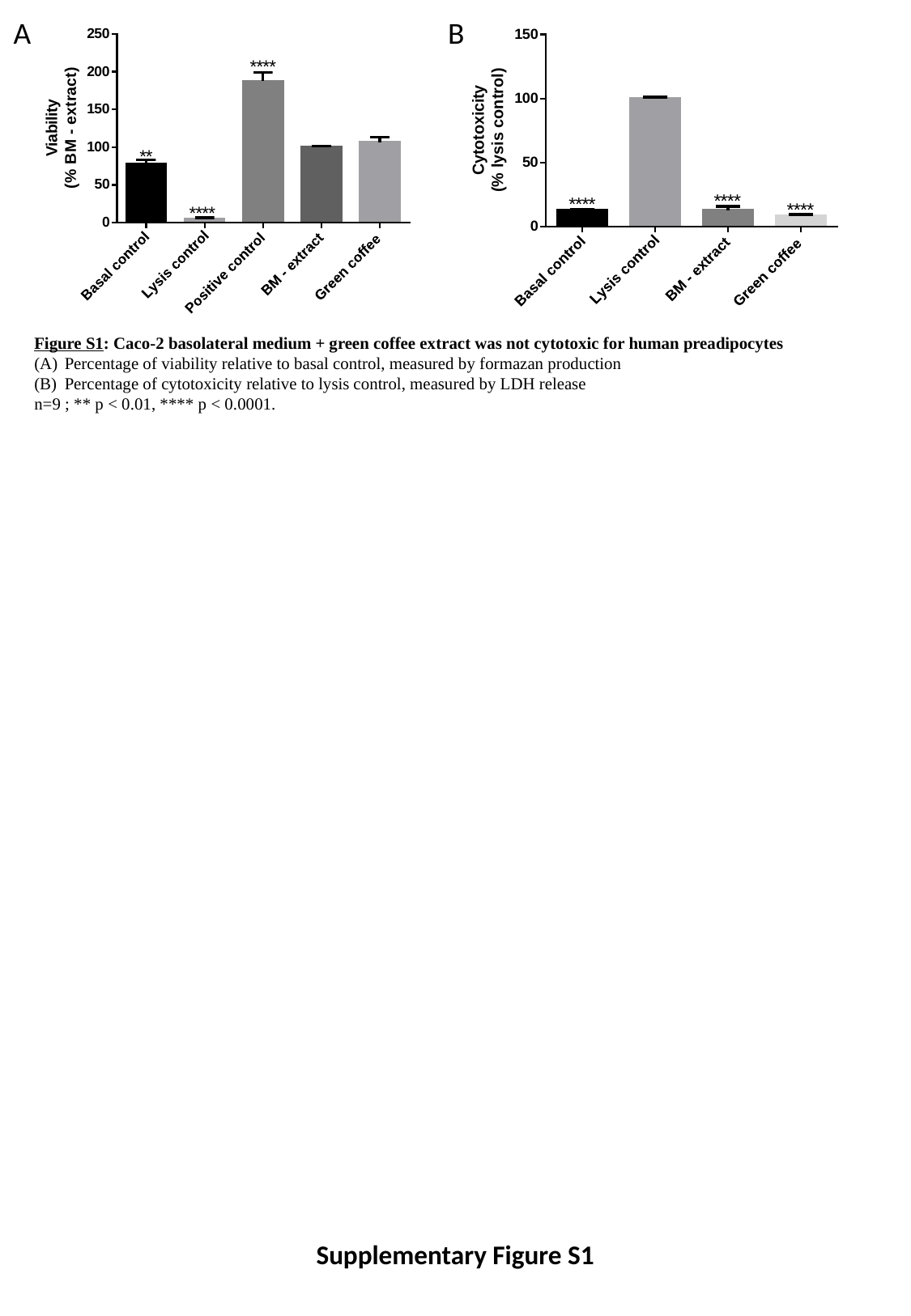

A
B
Figure S1: Caco-2 basolateral medium + green coffee extract was not cytotoxic for human preadipocytes
Percentage of viability relative to basal control, measured by formazan production
Percentage of cytotoxicity relative to lysis control, measured by LDH release
n=9 ; ** p < 0.01, **** p < 0.0001.
Supplementary Figure S1

## Slide 4
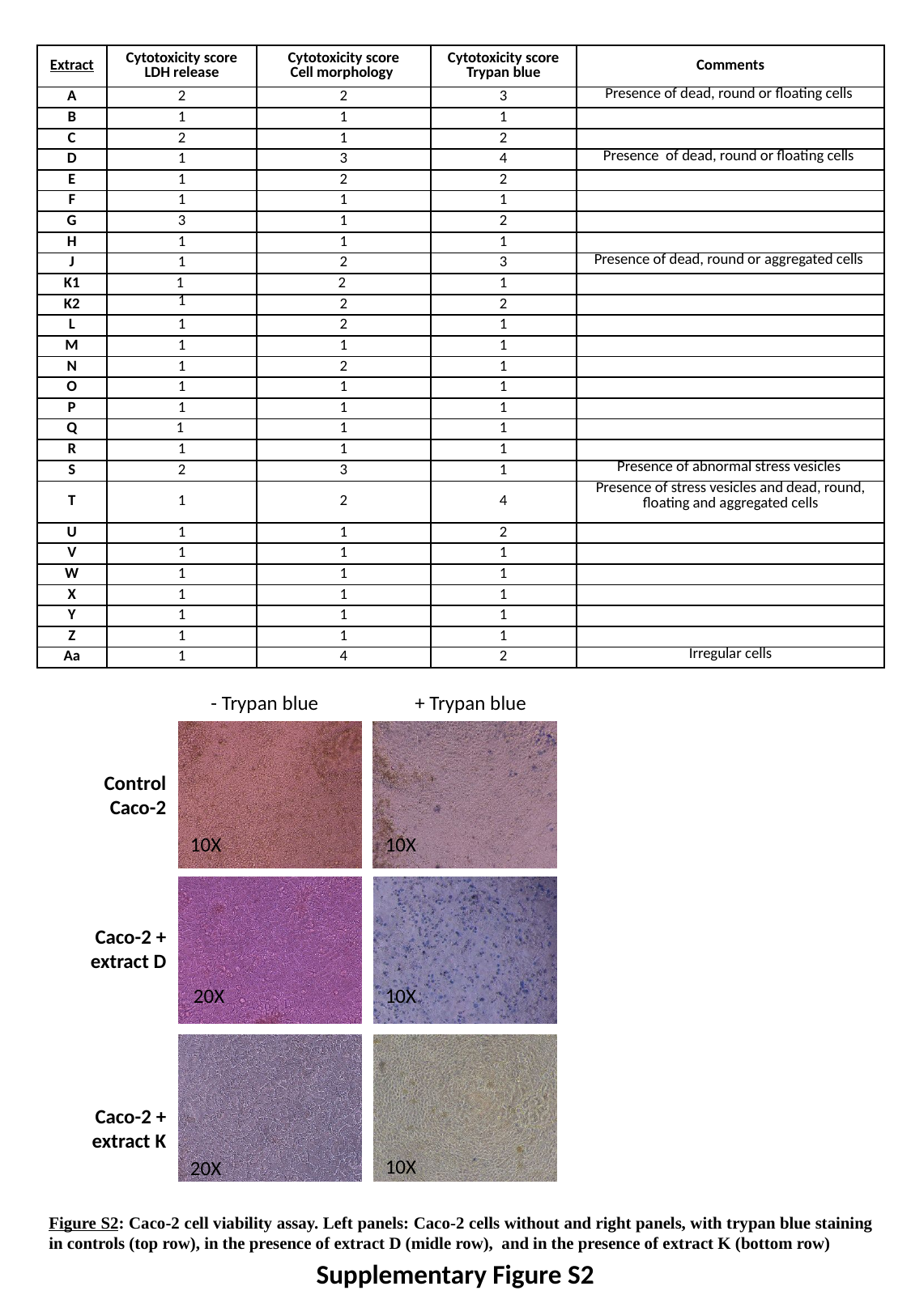

| Extract | Cytotoxicity score LDH release | Cytotoxicity score Cell morphology | Cytotoxicity score Trypan blue | Comments |
| --- | --- | --- | --- | --- |
| A | 2 | 2 | 3 | Presence of dead, round or floating cells |
| B | 1 | 1 | 1 | |
| C | 2 | 1 | 2 | |
| D | 1 | 3 | 4 | Presence of dead, round or floating cells |
| E | 1 | 2 | 2 | |
| F | 1 | 1 | 1 | |
| G | 3 | 1 | 2 | |
| H | 1 | 1 | 1 | |
| J | 1 | 2 | 3 | Presence of dead, round or aggregated cells |
| K1 | 1 | 2 | 1 | |
| K2 | 1 | 2 | 2 | |
| L | 1 | 2 | 1 | |
| M | 1 | 1 | 1 | |
| N | 1 | 2 | 1 | |
| O | 1 | 1 | 1 | |
| P | 1 | 1 | 1 | |
| Q | 1 | 1 | 1 | |
| R | 1 | 1 | 1 | |
| S | 2 | 3 | 1 | Presence of abnormal stress vesicles |
| T | 1 | 2 | 4 | Presence of stress vesicles and dead, round, floating and aggregated cells |
| U | 1 | 1 | 2 | |
| V | 1 | 1 | 1 | |
| W | 1 | 1 | 1 | |
| X | 1 | 1 | 1 | |
| Y | 1 | 1 | 1 | |
| Z | 1 | 1 | 1 | |
| Aa | 1 | 4 | 2 | Irregular cells |
- Trypan blue
+ Trypan blue
Control Caco-2
10X
10X
Caco-2 + extract D
20X
10X
Caco-2 + extract K
10X
20X
Figure S2: Caco-2 cell viability assay. Left panels: Caco-2 cells without and right panels, with trypan blue staining in controls (top row), in the presence of extract D (midle row),  and in the presence of extract K (bottom row)
Supplementary Figure S2

## Slide 5
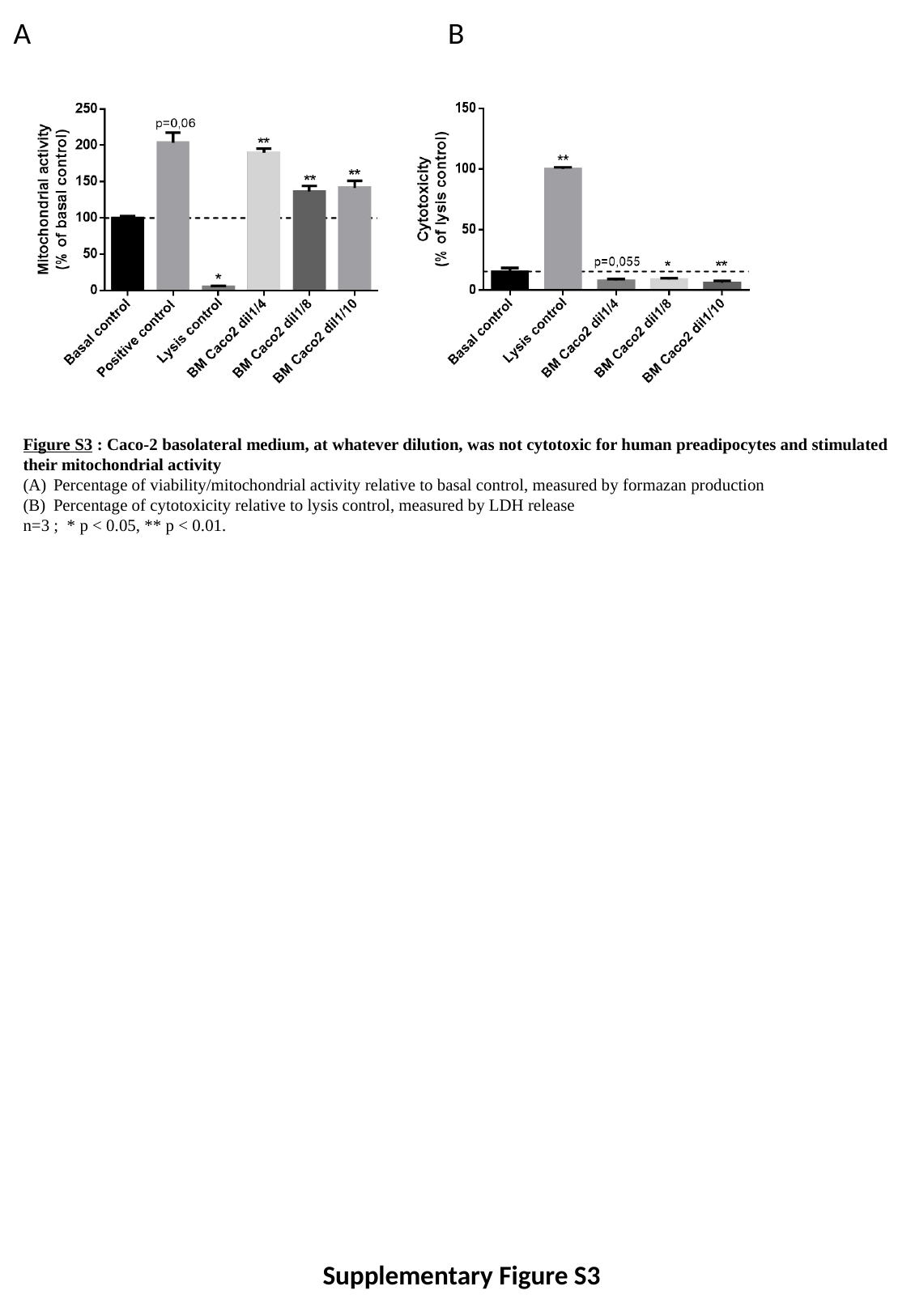

A
B
Figure S3 : Caco-2 basolateral medium, at whatever dilution, was not cytotoxic for human preadipocytes and stimulated their mitochondrial activity
Percentage of viability/mitochondrial activity relative to basal control, measured by formazan production
Percentage of cytotoxicity relative to lysis control, measured by LDH release
n=3 ; * p < 0.05, ** p < 0.01.
Supplementary Figure S3

## Slide 6
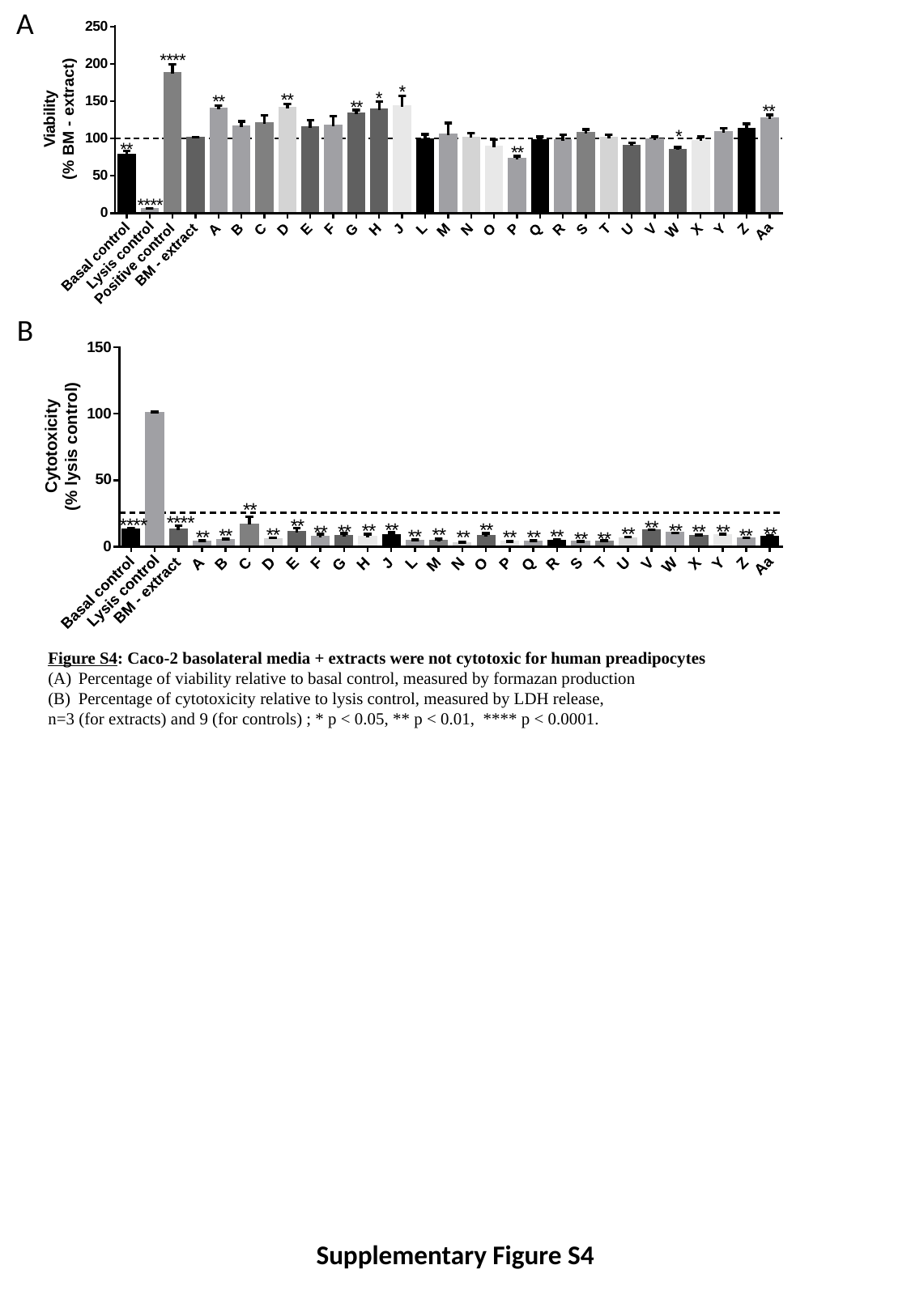

A
B
Figure S4: Caco-2 basolateral media + extracts were not cytotoxic for human preadipocytes
Percentage of viability relative to basal control, measured by formazan production
Percentage of cytotoxicity relative to lysis control, measured by LDH release,
n=3 (for extracts) and 9 (for controls) ; * p < 0.05, ** p < 0.01, **** p < 0.0001.
Supplementary Figure S4

## Slide 7
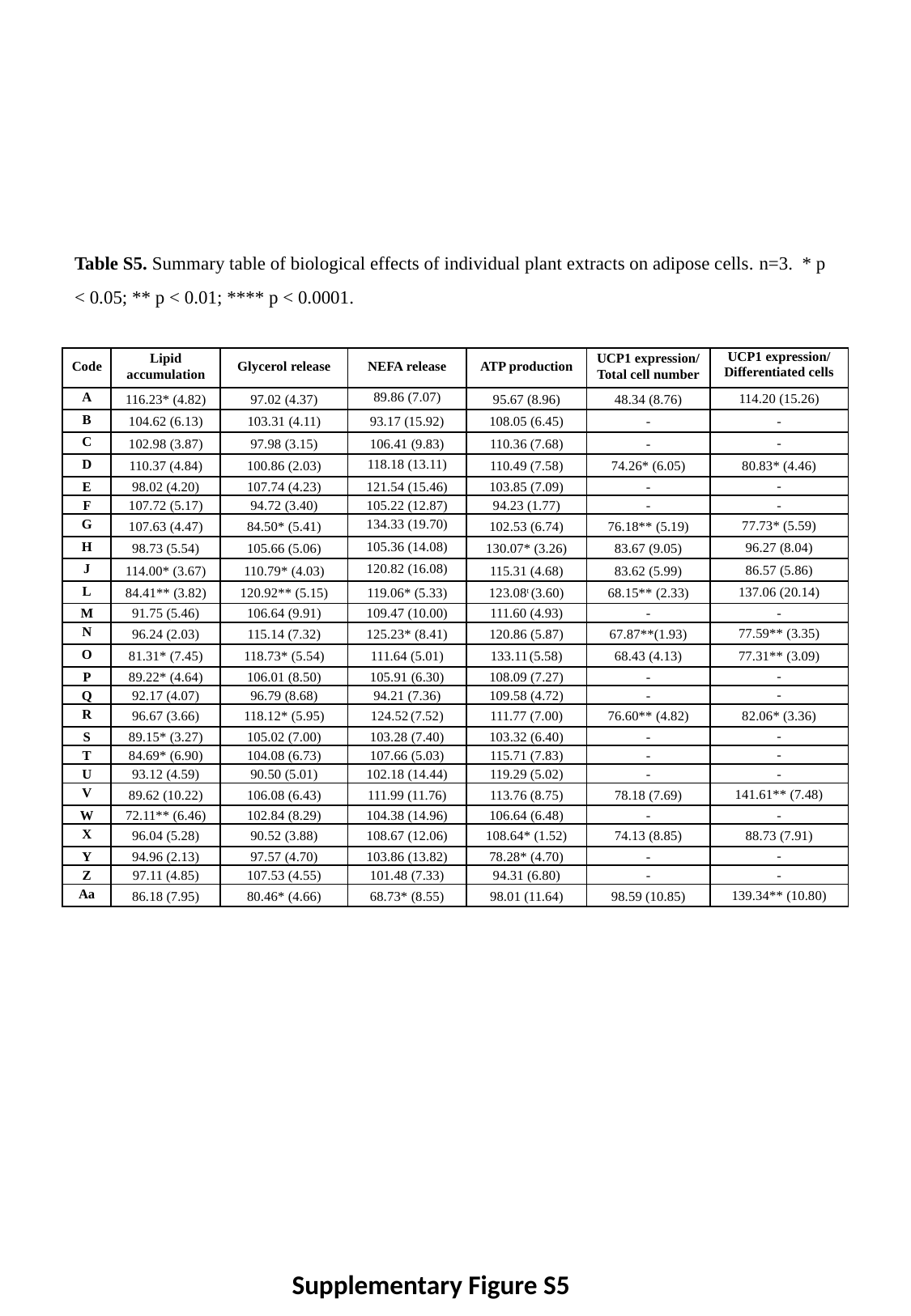

Table S5. Summary table of biological effects of individual plant extracts on adipose cells. n=3. * p < 0.05; ** p < 0.01; **** p < 0.0001.
| Code | Lipid accumulation | Glycerol release | NEFA release | ATP production | UCP1 expression/ Total cell number | UCP1 expression/ Differentiated cells |
| --- | --- | --- | --- | --- | --- | --- |
| A | 116.23\* (4.82) | 97.02 (4.37) | 89.86 (7.07) | 95.67 (8.96) | 48.34 (8.76) | 114.20 (15.26) |
| B | 104.62 (6.13) | 103.31 (4.11) | 93.17 (15.92) | 108.05 (6.45) | - | - |
| C | 102.98 (3.87) | 97.98 (3.15) | 106.41 (9.83) | 110.36 (7.68) | - | - |
| D | 110.37 (4.84) | 100.86 (2.03) | 118.18 (13.11) | 110.49 (7.58) | 74.26\* (6.05) | 80.83\* (4.46) |
| E | 98.02 (4.20) | 107.74 (4.23) | 121.54 (15.46) | 103.85 (7.09) | - | - |
| F | 107.72 (5.17) | 94.72 (3.40) | 105.22 (12.87) | 94.23 (1.77) | - | - |
| G | 107.63 (4.47) | 84.50\* (5.41) | 134.33 (19.70) | 102.53 (6.74) | 76.18\*\* (5.19) | 77.73\* (5.59) |
| H | 98.73 (5.54) | 105.66 (5.06) | 105.36 (14.08) | 130.07\* (3.26) | 83.67 (9.05) | 96.27 (8.04) |
| J | 114.00\* (3.67) | 110.79\* (4.03) | 120.82 (16.08) | 115.31 (4.68) | 83.62 (5.99) | 86.57 (5.86) |
| L | 84.41\*\* (3.82) | 120.92\*\* (5.15) | 119.06\* (5.33) | 123.08t (3.60) | 68.15\*\* (2.33) | 137.06 (20.14) |
| M | 91.75 (5.46) | 106.64 (9.91) | 109.47 (10.00) | 111.60 (4.93) | - | - |
| N | 96.24 (2.03) | 115.14 (7.32) | 125.23\* (8.41) | 120.86 (5.87) | 67.87\*\*(1.93) | 77.59\*\* (3.35) |
| O | 81.31\* (7.45) | 118.73\* (5.54) | 111.64 (5.01) | 133.11 (5.58) | 68.43 (4.13) | 77.31\*\* (3.09) |
| P | 89.22\* (4.64) | 106.01 (8.50) | 105.91 (6.30) | 108.09 (7.27) | - | - |
| Q | 92.17 (4.07) | 96.79 (8.68) | 94.21 (7.36) | 109.58 (4.72) | - | - |
| R | 96.67 (3.66) | 118.12\* (5.95) | 124.52 (7.52) | 111.77 (7.00) | 76.60\*\* (4.82) | 82.06\* (3.36) |
| S | 89.15\* (3.27) | 105.02 (7.00) | 103.28 (7.40) | 103.32 (6.40) | - | - |
| T | 84.69\* (6.90) | 104.08 (6.73) | 107.66 (5.03) | 115.71 (7.83) | - | - |
| U | 93.12 (4.59) | 90.50 (5.01) | 102.18 (14.44) | 119.29 (5.02) | - | - |
| V | 89.62 (10.22) | 106.08 (6.43) | 111.99 (11.76) | 113.76 (8.75) | 78.18 (7.69) | 141.61\*\* (7.48) |
| W | 72.11\*\* (6.46) | 102.84 (8.29) | 104.38 (14.96) | 106.64 (6.48) | - | - |
| X | 96.04 (5.28) | 90.52 (3.88) | 108.67 (12.06) | 108.64\* (1.52) | 74.13 (8.85) | 88.73 (7.91) |
| Y | 94.96 (2.13) | 97.57 (4.70) | 103.86 (13.82) | 78.28\* (4.70) | - | - |
| Z | 97.11 (4.85) | 107.53 (4.55) | 101.48 (7.33) | 94.31 (6.80) | - | - |
| Aa | 86.18 (7.95) | 80.46\* (4.66) | 68.73\* (8.55) | 98.01 (11.64) | 98.59 (10.85) | 139.34\*\* (10.80) |
Supplementary Figure S5

## Slide 8
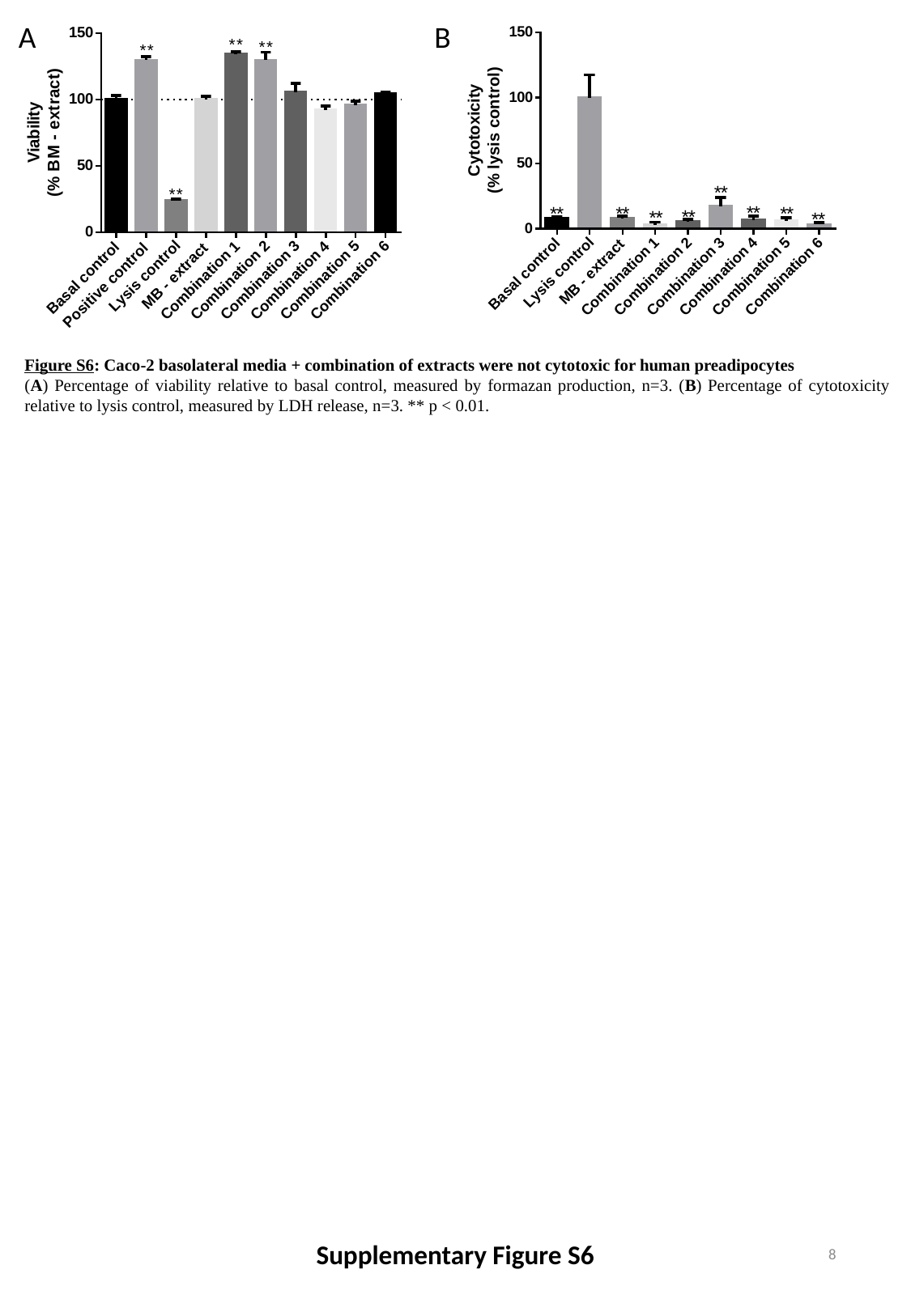

A
B
Figure S6: Caco-2 basolateral media + combination of extracts were not cytotoxic for human preadipocytes
(A) Percentage of viability relative to basal control, measured by formazan production, n=3. (B) Percentage of cytotoxicity relative to lysis control, measured by LDH release, n=3. ** p < 0.01.
8
Supplementary Figure S6
